# The accumulation of metals, PAHs and alkyl PAHs in the roots of *Echinacea purpurea*

**DOI:** 10.1371/journal.pone.0208325

**Published:** 2018-12-06

**Authors:** Travers R. Pretorius, Christiane Charest, Linda E. Kimpe, Jules M. Blais

**Affiliations:** Department of Biology, University of Ottawa, Ottawa, ON, Canada; Universidade de Coimbra, PORTUGAL

## Abstract

We examined the accumulation of polycyclic aromatic hydrocarbons (PAHs), alkyl PAHs, and toxic metals in soils by the roots of *Echinacea purpurea* (L.) Moench, in a 20-week greenhouse study and a 2-year field study. In the greenhouse study, inoculation by arbuscular mycorrhizal fungus (AMF), *Rhizoglomus intraradices* (N.C. Schenck & G.S. Sm.). increased the first order accumulation rates (k_1_) for PAHs by 10-fold, though had no effect on the bioaccumulation rates of toxic metals. In the greenhouse study, PAHs concentrations in soil increased over time with AMF inoculation, suggesting AMF promote ‘solvent depletion’ in soils by enhancing absorption of minerals and carbon by roots, concentrating the more hydrophobic PAHs in the residual soil. Under field conditions, contaminant concentrations in soils remained unchanged over the 2-year duration of the study. Despite this, all contaminants in *E*. *purpurea* roots increased significantly, as a result of a long term extraction of contaminants by plants from soil and a reduction in soil volume as a result of plant growth. First order accumulation rates by roots were inversely correlated to log K_ow_ for the PAHs and alkyl PAHs, indicating that accumulation is inversely related to the compound’s hydrophobicity. This study is the first to our knowledge to assess the accumulation of alkyl PAHs by roots, with implications for soil bioremediation by plants because alkyl PAHs are a major source of petrogenic contamination in soils.

## Introduction

Contamination of soil by organic and inorganic pollutants is a growing problem due to industrialization, intensive agriculture, and the widespread use of xenobiotics [1–3]. Exposure to these contaminants poses a significant risk to human and ecological health [4,5] and the need for remediating soils is urgent. To date, bioremediation has been recognized as cost-effective, reliable and promising technology for reclaiming contaminated soils [6–8].

The PAHs and alkyl PAHs have been the focus of many bioremediation programs due to their ubiquitous presence in contaminated soils, their acute toxicity, carcinogenicity, mutagenicity, teratogenicity, and their effects on endocrine function [9–13]. PAHs consist of two to seven fused benzene rings that are arranged in various structural configurations. Alkyl PAHs usually have one to four saturated carbon atoms and can produce many different structural isomers and homologs for each hydrocarbon family. Their hydrophobic nature leads to increased accumulation and enrichment in soils, which is cause for remediation of contaminated sites.

The toxic metals we consider here were chosen based on the U.S. EPA’s 13 metals and metalloids listed as priority pollutants. Although many metals are essential for organisms many of them can be toxic at higher concentrations causing oxidative stress, disrupting enzyme activity or interfering with the structure and function of proteins. Unlike organic contaminants, metals cannot be degraded.[14–16] In order for plants to detoxify their environment, they either release root exudates to chelate metals, or produce intracellular phytochelatins and metallothioneins which possess a high affinity for metals that are then sequestered in their vacuoles [14,17]. Taking advantage of this process can create a viable option for remediating contaminated soils.

Plants can facilitate enhanced degradation of organics and increased absorption of inorganics by creating favourable conditions for microbial degradation, and accessing contaminants through their root system [5,18]. Plant-microbe associations are gaining considerable attention with enhanced remediation, positive effects on plant establishment and survival of plants in contaminated soil [3,19–21]. In particular, arbuscular mycorrhizal fungi (AMF) may enhance contaminant accumulation from soils due to their extraradical hyphae having access to fine soil pores that are unavailable to plant roots [22].

AMF are complex, ancient organisms that are ubiquitous in nature, found in 80–90% of all terrestrial plants [23]. They play fundamental roles for plants, as by increasing nutrient uptake, improving tolerance to environmental stress, and influencing soil microbial communities [24]. AMF have also been shown to increase uptake of both organic and inorganic contaminants from soil. Cheng et al. [25] and Debiane et al. [26] showed that alfalfa roots colonized by AMF had increased PAH accumulation and Gao et al. [22] reported the same in ryegrass. Here, we examined the potential for *Echinacea purpurea* (L.) Moench (hereafter *E*. *purpurea*) plants inoculated or not by an AMF species to accumulate two classes of contaminants: (1) polycyclic aromatic hydrocarbons (PAHs) and their alkyl homologs in soils; and (2) toxic metals. To our knowledge this is the first study that includes the uptake of alkyl homologues with parent PAHs and metals in plant roots.

We selected *E*. *purpurea* for this study based on work by Araim et al. [27] for its capacity to synthesize secondary compounds in response to biotic stress, and Liu et al. [28] for its effectiveness in remediating total PAHs contaminated soil among 14 ornamental species. In this study by Liu et al. [28] *E*. *purpurea* reduced total petroleum hydrocarbons by 46.74% in 10,000 mg kg^-1^ total petroleum hydrocarbon contaminated soil after a 30-day pot culture experiment. This was only second to Fawn (*Festuca arundinacea* Schreb), which reduced total petroleum hydrocarbons by 49.42%. Further advantages of using *E*. *purpurea* were its positive response to an available inoculum of *Rhizoglomus intraradices*, its aesthetic for use in an urban environment, its drought tolerance and its fibrous root system that allows greater contact with soil compared to a taproot [29]. *E*. *purpurea* has also been favoured due to its low production cost, increased biomass per hectare, and ease of cultivation relative to the other varieties, *E*. *pallida* and *E*. *angustifolia* [30,31]. We predicted that PAHs, alkyl PAHs and toxic metals will show increased uptake and content in AMF colonized *E*. *purpurea* roots and shoots. The aim of this study was to quantify the accumulation rates of contaminants by *E*. *purpurea* for the remediation of soils contaminated with PAHs, alkyl PAHs and toxic metals.

## Materials and methods

### Greenhouse study

A 20-week greenhouse experiment was conducted using a factorial block design (1 plant sp. x 2 M x 2 harvests) with *E*. *purpurea*, inoculated with or without *Rhizoglomus intraradices*, DAOM 181602, Premier Tech, Rivière-du-Loup, QC, and grown in homogenized soil samples collected from ten test pits (1m deep) from Victoria Island, Ottawa, ON (45° 25’ 15” N, 75° 42’ 50” W). We obtained permission for our field experiment from the National Capital Commission who is responsible for this field site.

The *E*. *purpurea* seeds (Ontario Seed Company, Waterloo, ON) and pots (12.5 x 12.5 x 15 cm) were surface sterilized by a 10% (v/v) solution of NaOCl for 10 min, and rinsed with sterile distilled water before sowing. The fungal inoculum or a control substrate was integrated as a 3-cm thick substrate layer on top of 1 L of soil, and then covered with soil as determined by Audet and Charest [32]. Eight seeds were sown ~1 cm deep in the soil mixture and thinned after two weeks to one plant per pot as determined by Araim et al. [27]. Replicates of bare soil, labelled as control soil, were also prepared to compare the pre- and post-experimental soil.

The greenhouse conditions were maintained with a photoperiod of 16:8 L:D provided by natural light and high-pressure sodium lamps (PL Light Systems, Beamsville, ON, Canada), a day/night temperature regime of 27°C/23°C and a 40% relative humidity. The average light intensity (408 μmol s^-1^ m^-2^) was measured using a light meter with a quantum sensor (LiCor LI-250A and LI -190SA, Lincoln, NE). Plants were watered with dH_2_O on a daily basis as required, without water leaking through the pots, then fertilized after the 3^rd^ week following germination with 20 mL of ½ ammonium nitrate type Long Ashton Nutrient Solution (LANS; [33]), once a week for two weeks (the 4^th^ and 5^th^ weeks), then fertilized with the full LANS (50 mL at the 6^th^ week, and 100 mL) until harvest. Plants were harvested after 10 (n = 3) and 20 (n = 5) weeks of growth.

### Field study

A factorial block design (1 plant sp. x 2 M x 2 harvests) field study was also conducted (August 2013 to August 2014) using 6-week grown old *E*. *purpurea* plantlets from the greenhouse, pre-inoculated or not by *R*. *intraradices*, 6-weeks prior to being grown in tilled soil on Victoria Island, Ottawa. For nearly a century, from the late 1800’s until 1960 Victoria Island has served as an industrial area with foundries, a pulp and paper mill, a scrap iron company, a calcium carbide factory, and housed military buildings and offices. With these historical industrial activities the site was exposed to both organic and inorganic pollutants that have become weathered over time. Utilizing *E*. *purpurea*, a plant species shown to effectively reduce total petroleum hydrocarbons [28] and form mutualistic relationship with AMF [27], the present study aimed to enhance the uptake of both organic and inorganic pollutants from the soil of a former industrial site.

The plot was of 6 m x 4 m and contained 5 blocks. Each block was 1.3 m x 1.2 m and comprised three rows; control soil, non-AMF treatment and AMF treatment, each 0.3 m wide with spacing of 0.2 m between rows (S1 Fig). Compost was applied as a 0.1 m thick layer and covered with an equally thick layer of natural cedar mulch. Industrial contaminated soils from Victoria Island, Ottawa, were provided by the National Capital Commission. Control soil, was a row of bare soil covered with natural cedar mulch in each of the blocks.

Plants were started in the greenhouse prior to being transplanted in the field to ensure survival from seed predators. Plantlets of *E*. *purpurea* were grown from seeds in autoclaved PRO-MIX soil for 6 weeks and sown with or without AMF propagules of *R*. *intraradices* before being transplanted into the field site. The pots were prepared in the same manner as in the greenhouse experiment with the only difference of the pot size (0.5 L). The plants were harvested after 10 weeks of growth in the field (Year 1). The following year (Year 2), the second harvest occurred after 48 weeks. Collection and sample preparation were completed in the same manner as described in the greenhouse study. In Year 2, due to the large plant size, subsamples of shoots and roots from each plant, and soils were taken and analyzed (n = 5 plants or soil per treatment).

### Percent organic matter

Percent organic matter (%OM) was determined using sequential loss on ignition (LOI) method [34]. The determination of %OM was done by oxidizing organic matter at 550°C to CO_2_ and ash using a muffle furnace (Barnstead|Thermoline, 30400 Furnace). The weight loss was determined by weighing the samples on an analytical balance (Mettler Toledo, AG104) before and after heating. The %OM was determined using LOI_550_ (1) and percent carbonate content was determined using LOI_950_ (2):

$$\left(\frac{DW105-DW550}{DW105}\right)\times 100$$ (1)

$$\left(\frac{DW550-DW950}{DW105}\right)\times 100$$ (2)

### Assessment of arbuscular mycorrhizal root colonization

Root samples from three plants (n = 10, number of sections from each plant) were prepared according to Dalpé [35]. Fresh root samples were washed, patted dry and stained using a 0.02% aniline blue dye solution (0.5 g of aniline blue, 500 mL of glycerol, 450 ml of dH_2_O, and 50 ml of 1% HCl.). Root segments were examined at 10x and 40x magnification using a compound microscope. AMF colonization was estimated by determining the counts and relative density of the diverse fungal structures (hyphae, vesicles or spores) [36].

### Environmental site investigation

In an environmental site investigation done in 2003, 43 boreholes were dug on the Victoria Island site. Analysis of the soil samples for metals and PAHs revealed elevated levels of copper, lead, benzo(a)anthracene, benzo(a)pyrene, benzo(b)fluoranthene, benzo(k)fluoranthene, indeno(1,2,3-cd)pyrene, and phenanthrene. The elevated levels of both metals and PAHs exceeded guidelines set out by Canadian Council of Ministers of the Environment (CCME) for residential/parkland and commercial/industrial land (Table 1).

**Table 1:** Victoria Island soil sample analytical results from 2004 environmental investigation.

				Borehole # and Depth				
	CCME Guidelines			10	11	12	13	14
	Residential/Parkland	Industrial/ Commercial		0–0.6m	0–0.4m	0–0.6m	0–0.6m	0–0.6m
**Copper**	**63**	**91**		25	**80**	15	15	15
**Lead**	**140**	**260**		**160**	**210**	25	160	130
**Benzo(a)anthracene**	**1**	**10**		0.6	**5.5**	**1.4**	0.04	0.16
**Benzo(a)pyrene**	**0.7**	**0.7**		0.66	**5**	**1.6**	0.04	0.2
**Benzo(b)fluoranthene**	**1**	**10**		0.48	**3.8**	**1.3**	0.04	0.18
**Benzo(k)fluoranthene**	**1**	**10**		0.46	**3.3**	**1.1**	0.04	0.08
**Indeno(1,2,3-cd)pyrene**	**1**	**10**		0.3	**1.9**	0.8	0.02	0.1
**Phenanthrene**	**5**	**50**		0.46	**9.2**	2.4	0.04	0.08

Note: all units are micrograms per gram (μg/g)

CCME Canadian Council of Ministers of the Environment "Canadian Environmental Quality Guidelines" 1999, Revised in part 2002. Chapter 7: Canadian Coil Quality Guidelines for the Protection of Environmental and Human Health (Residential/Parkland and Commercial Land Uses)

### Analysis of polycyclic aromatic hydrocarbons (PAHs), and alkyl PAHs

PAH concentrations (ng g^-1^) were multiplied by the respective mass of root and shoot for their content and measured as PAH or alkyl PAH content (ng). PAHs and alkyl PAHs in soil were measured as concentration (ng g^-1^). All 16 US EPA priority PAHs and 21 alkyl PAHs analyzed are listed in S1 Table.

Soil and plant samples were analyzed using gas chromatography and mass spectrometry. All plant and soil samples were homogenized with elemental copper and Agilent brand Hydromatrix. Samples were then spiked with known concentrations of ^13^C-labeled PAHs (Cambridge Isotope Laboratories, Tewksbury, MA, USA) and extracted using accelerated solvent extraction module (ASE-350, Dionex Corporation, Sunnyvale, CA, USA) at 140°C using 1:1 hexane: acetone mixture following methods USEPA Method 3640A. Extracts then underwent liquid-liquid extraction with hexane to remove organic compounds from the co-extracted water. Following liquid-liquid extraction, extracts were concentrated using a TurboVap (Biotage, Charlotte, NC, USA) under a gentle nitrogen stream. Clean up with USEPA Method 3630C was adapted for use on 6ml (1g) Superclean TM LC-Si solid-phase extraction cartridges. Samples were further concentrated to approximately 1 mL, which was the final extract volume for all samples. Internal standard p-terphenyl-d14 was added to all final extracts. Analysis of the final extract was done by injecting 1μL of sample into a gas chromatograph (Agilent 6890) and mass spectrometer (Agilent 5973). Separation was completed on a DB5-MS 30 m x 0.25 mm x 0.25 μm column with H_2_ as the carrier gas. Using single ion monitoring and the ^13^C-labeled PAHs, 16 US EPA priority PAHs and 21 alkyl PAHs were analyzed and quantified. All samples were blank corrected to remove background contamination, and replicate extractions were carried out on Standard Reference Material (SRM) 1941b –Organics in Marine Sediment from the National Institute of Standards and Technology (Gaithersburg, MD, USA).

### *k*_*1*_ accumulation rate of PAHs in roots

The rate of accumulation for PAH compounds roots were determined using the following formula that was modified from Gobas and Morrison [37]:

$$k_{1}=\frac{\left(dCr/dt\right)}{Cs}$$

Where *k*_1_ is the rate constant for the accumulation of the PAH compound in units day^-1^, dCr the initial change in concentration of PAH compound in roots, dt the time interval (70 days) and Cs the soil concentration.

### Metal analyses

The toxic metals that were considered here were chosen based on the U.S. EPA’s 13 metals and metalloids listed as priority pollutants. Metal concentrations (mg kg^-1^) were multiplied by the respective mass (kg) of root and shoot to determine metal content (mg). Fresh mass was determined from Muntean et al. [38,39], as whole plants were too large to collect from field site. Metals in soil were measured as concentration (mg kg^-1^ dry mass). The treatments were taken in three and five replicates for the greenhouse and field study, respectively.

Before and after the growth period, metals were determined using ICP-MS. Samples of root, shoot, and soil were individually digested using a hot nitric acid solution following a modified version of US-EPA method 3050. This was done by digesting freeze-dried samples in 10 mL of 1:1 HNO_3_ and HCl and heated on a graphite block (DigiPREP MS block digestion system, SCP Science, QB, Canada) for 30 minutes at 90°C. Once cooled to room temperature, 30% H_2_O_2_ was added and then heated at 90°C for 3 hours. Samples were then diluted with ultra pure Milli-Q H_2_O up to 50 mL mark. All samples were filtered to remove any particulate. Filtered samples were then diluted 10:1 using ultra pure Milli-Q H_2_O. In the same manner, standard reference material (Buffalo River Sediment #8704) from the National Institute of Standards and Technology (NIST, USA) and blanks consisting of HNO_3_ and HCl were also prepared to ensure quality and accuracy of the metal analysis. The digested extracts were then analyzed using an Agilent ICP-MS 7700 series following US-EPA method 200.8., which was run using a reactive gas for the removal of known interferences in a complex unknown mixture without loss of sensitivity.

### Statistical analyses

One and two-way ANOVA’s were performed for all the data and comparisons among means were performed using Tukey’s test. An independent t-test was used for AMF colonization and simple linear regressions were used for k_1_ accumulation rates. Shapiro-Wilk and Levene’s tests were used to verify the normality of distribution and the homogeneity of variance respectively. The data were log transformed as required to meet the assumptions of parametric analysis. All statistical analyses were done using R statistical software (version 3.2.3).

## Results

### Greenhouse study

During the investigation (2012–2014), we characterized the pre-experimental soil. The homogenized soil from Victoria Island showed an average pH of 6.77, and an organic matter content of 4.01%. Mineral soil composition in the greenhouse with the most abundant elements were Ca, P, and Ti (42,400 mg kg^-1^, 1,040 mg kg^-1^, 631 mg kg^-1^, respectively), and the least abundant were Fe (13.8 mg kg^-1^), Na (0.315 mg kg^-1^) and Sr (0.0899 mg kg^-1^).

Overall, mycorrhizal colonization was found in both the inoculated and non-inoculated roots from the greenhouse experiment. However, the inoculated plants consistently had significantly higher colonization counts of hyphae, vesicles and spores relative to the non-inoculated soil (*t*-test p<0.01, Table 2).

**Table 2:** AMF root colonization count and density measurements in the greenhouse study.

		Count			Density (mm^-1^)		
Week	Treatment	Hyphae	Vesicles	Spores	Hyphae	Vesicles	Spores
**10**	AMF	227.0 (35.3)	1128.7 (1002.1)	4.3 (1.2)	0.45(0.07)	2.26(2.00)	0.01(0.00)
	Non-AMF	92.7 (50.3)	80.0 (73.6)	0.3 (0.3)	0.1 (0.10)	0.16(0.15)	0.00(0.00)
**20**	AMF	71.3 (6.4)	623.67 (273.22)	6.7 (2.0)	0.14(0.01)	1.25(0.55)	0.01(0.00)
	Non-AMF	6.7 (3.3)	130.7 (54.7)	11.0 (9.0)	0.01(0.01)	0.26(0.11)	0.02(0.02)
**t-values and levels of significance**							
**10**	Treatment	2.2ns	1.0ns	3.2ns			
**20**	Treatment	8.9**	1.8ns	-.5ns			

Note: Each treatment is 150, 1cm root segments (10 per slide) observed under a compound microscope. Means (n = 3) and (SE) are shown for each treatment.

ns: not significant

* p<0.05

** p<0.01

*** p<0.001

Soil PAH concentrations were significantly affected by the different treatments (p = 0.002) over the course of the greenhouse experiment. PAH concentrations in soil ranged between 6,830 to 14,000 ng g^-1^, with control soil having the lowest concentration at week 10, while non-AMF soil had the highest at week 20.

**Fig 1:**
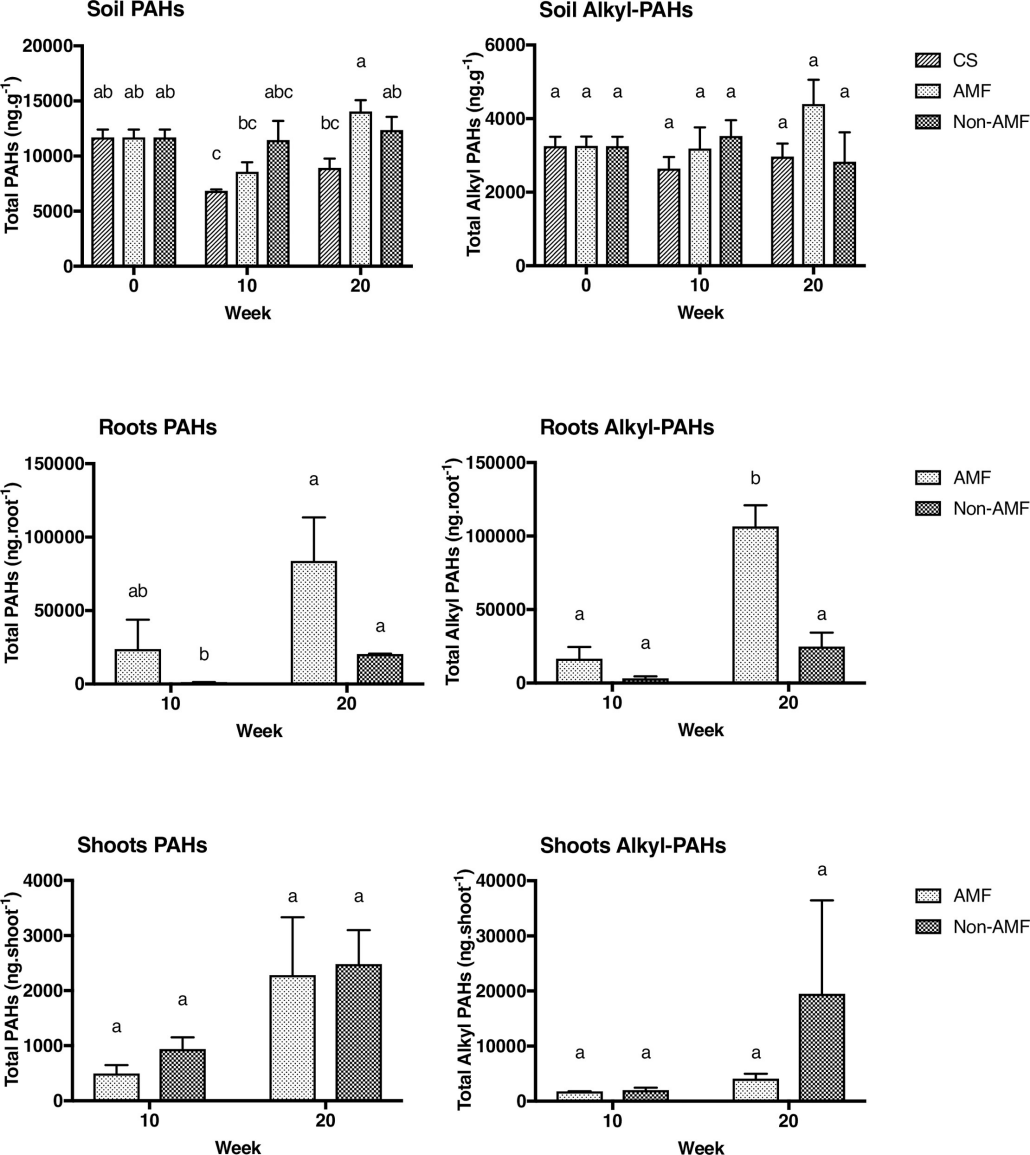
Mean (±SE) PAHs and alkyl-PAH concentrations soil (dry weight) and Total PAHs and Alkyl PAHs in roots (ng.root^-1^) and shoots (ng.shoot^-1^) of *E*. *purpurea* in the greenhouse (n = 3. Data were analyzed using a one-way ANOVA for roots and shoots. Different letters indicate significant differences according to Tukey’s post-hoc test. AMF: *E*. *purpurea* inoculated with *R*. *intraradices*, non-AMF: *E*. *purpurea* only.

**Fig 2:**
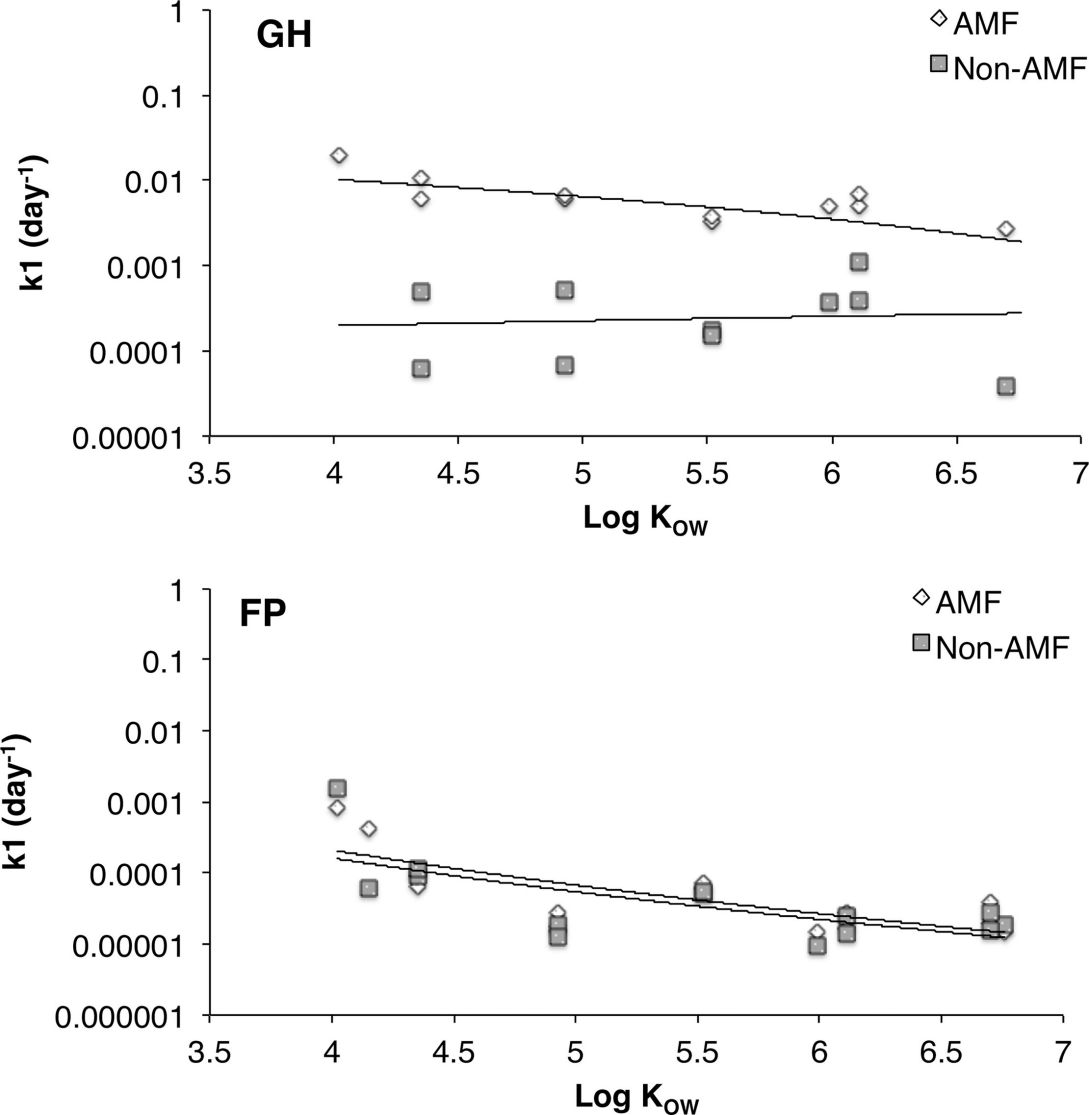
Uptake rates (k_1_) on a logarithmic scale measured in roots of arbuscular mycorrhizal fungi (AMF) and non-arbuscular mycorrhizal fungi (non-AMF) plants during week 10 (W10) (a) and year 1 (Y1) (b) of the greenhouse and field experiment plotted against the Log K_OW_’s of selected PAHs. In the greenhouse AMF roots show a significant negative relationship (slope = -0.17, r^2^ = -0.47, p = 0.014) while non-AMF roots showed no significance (slope = 0.0095, r^2^ = 0.00026, p = 0.97). In the field experiment, AMF roots showed a significant relationship (slope = -2.62, r^2^ = 0.36, p = 0.022) while non-AMF roots showed no significant relationship (slope = -1.80, r^2^ = 0.21, p = 0.098). Log K_OW_ are given for the following PAHs: acenaphthene, fluorene, phenanthrene anthracene, fluoranthene, pyrene, benz[a]anthracene, chrysene, benzo[b]fluoranthene, benzo[k]fluoranthene, benzo[a]pyrene, indeno[1,2,3- cd]pyrene, dibenz[a,h]anthracene, and benzo[g,h,i]perylene.

AMF inoculation in the greenhouse study increased the PAH accumulation in roots (p = 0.006), (Fig 1). Examination of AMF roots in the greenhouse study showed that they accumulated ~23 times more PAHs at week 10 and ~4 times more at week 20 compared to non-AMF roots, with no effect in shoots of *E*. *purpurea* (Fig 1, Fig 2).

**Fig 3:**
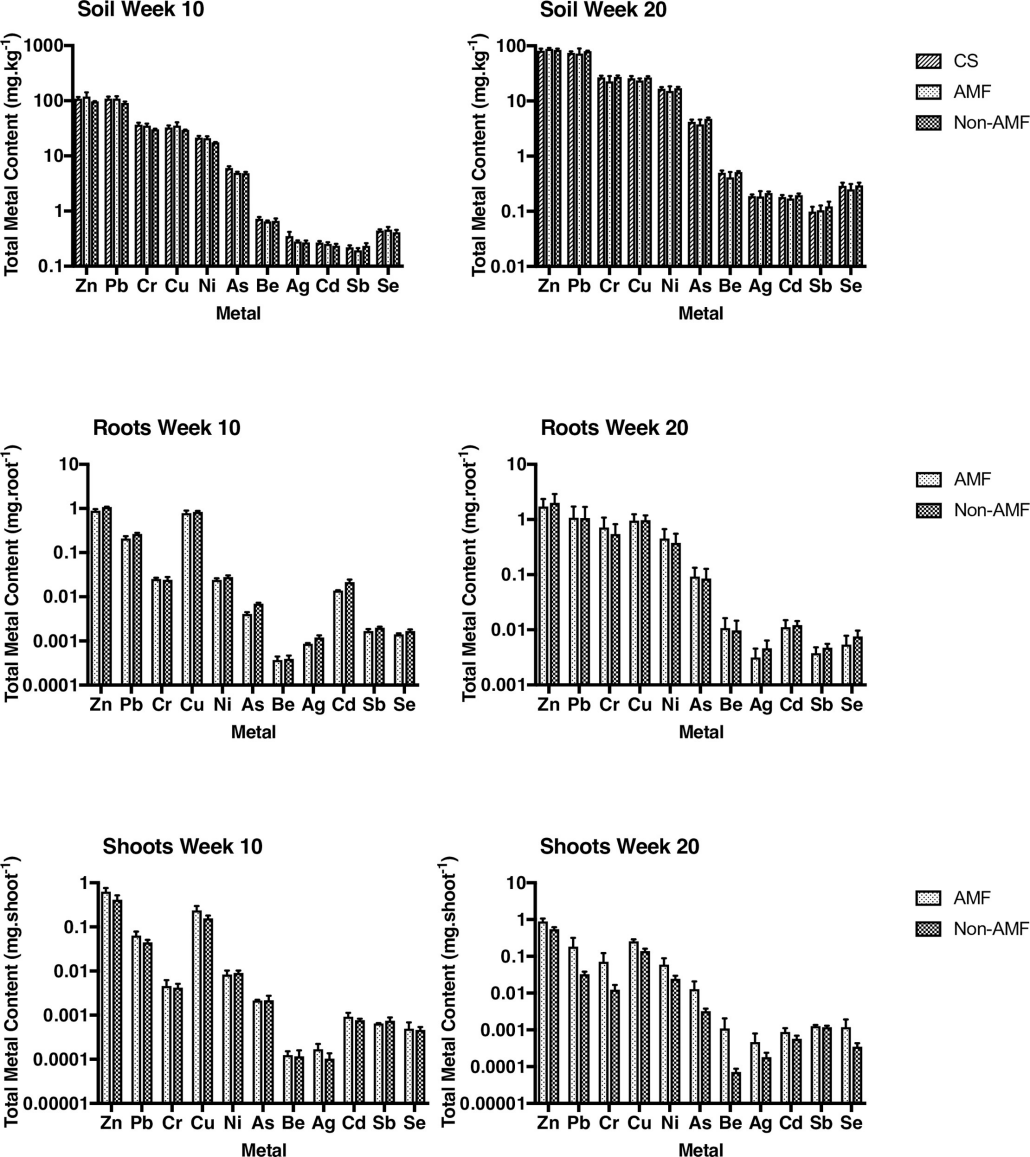
Mean (±SE) metal concentration (mg.kg^-1^) in soil and total metal content in roots (mg.root^-1^) and shoots (mg.shoot^-1^) in greenhouse samples from week 10 to week 20. Soil n = 5, roots and shoots each n = 10. AMF: *E*. *purpurea* inoculated with *R*. *intraradices*, non-AMF: *E*. *purpurea* only.

The alkyl PAHs accumulated to a greater extent in the roots and shoots despite PAHs being more abundant in the soil. The accumulation of alkyl PAHs in the roots was also significantly higher (p = 0.002) when exposed to the AMF (Fig 1, Fig 2). At week 20, alkyl PAHs had accumulated ~4.5 times more in AMF roots than non-inoculated roots. The accumulation of alkyl PAHs in shoots showed no significant difference between treatment groups in the greenhouse study. Also, k_1_ accumulation rates for PAHs in AMF roots showed a significant negative relationship (slope = -0.17, r^2^ = -0.47, p<0.05) with log K_OW_, indicating that accumulation rates decrease as hydrophobicity increases (Fig 3). Non-AMF mycorrhizal roots had ~10 x lower k_1_ values compared to AMF roots, and were not correlated with log K_OW_. These results clearly show an important role of AMF in enhancing accumulation of PAHs by roots.

**Fig 4:**
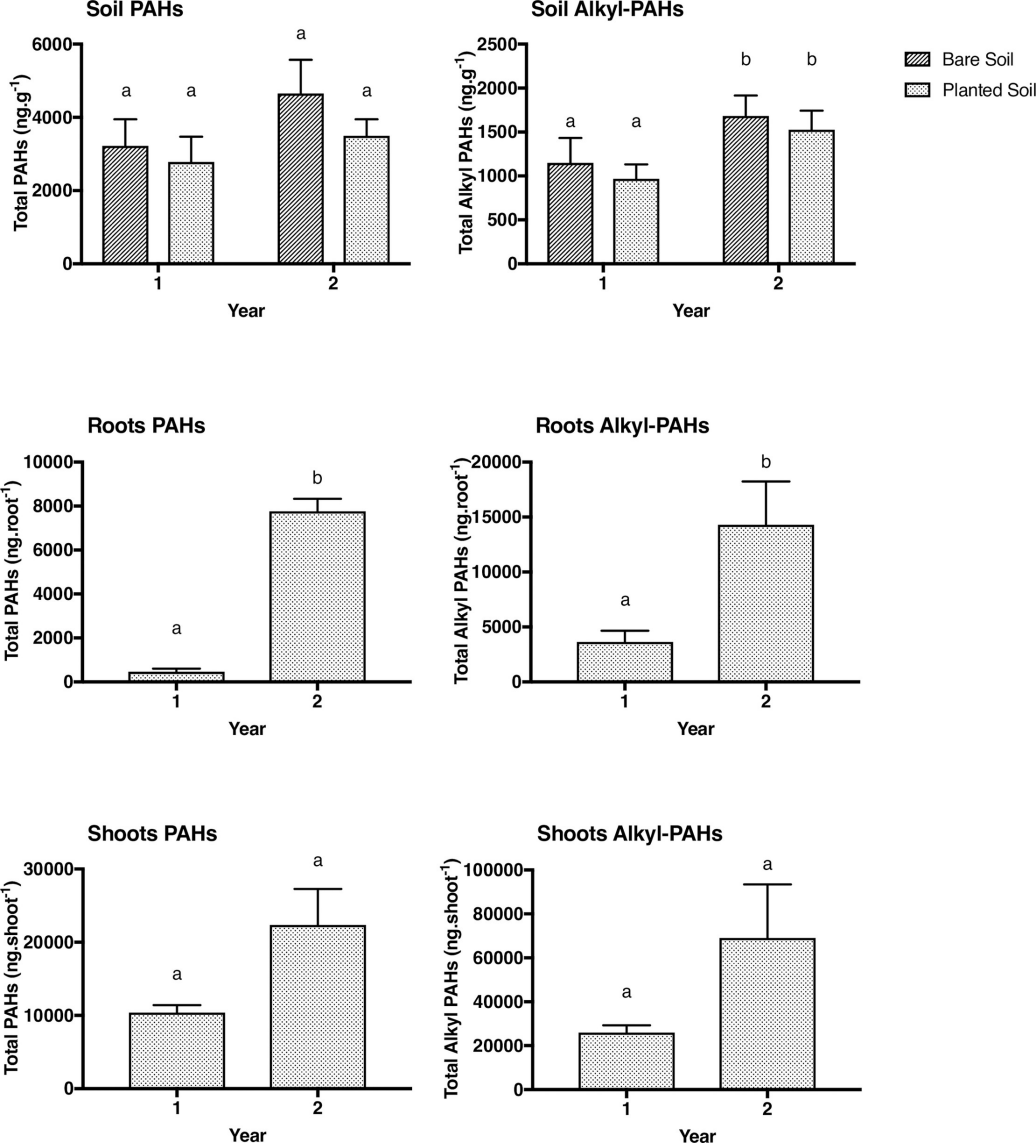
Mean (±SE) PAHs and alkyl-PAH concentrations soil (dry weight) and Total PAHs and Alkyl PAHs in roots (ng.root^-1^) and shoots (ng.shoot^-1^) (n = 10) of *E*. *purpurea* at the field site. Data were analyzed using a one-way ANOVA for roots and shoots. Different letters indicate significant differences according to Tukey’s post-hoc test.

Soil concentrations of Cu, Se, and Zn were significantly reduced in the greenhouse soil (p<0.05) from week 10 to week 20. Cd had a significant decrease in roots, whilst Cu significantly increased (p<0.05) over time. In the shoots, Cr significantly increased (p<0.05) (Fig 4) from week 10 to week 20.

### Field study

Mycorrhizal colonization was found in both the inoculated and non-inoculated roots in the field experiment. There was no significant difference between the inoculated and non-inoculated soil (*t*-test p>0.05, Table 3). Although, the inoculated plants consistently had higher colonization counts of hyphae, vesicles and spores relative to the non-inoculated soil.

**Table 3:** AMF root colonization count and density measurements at year 2 from field study.

	Count			Density (mm^-1^)		
Treatmentt	Hyphae	Vesicles	Spores	Hyphae	Vesicles	Spores
AMF	34.7 (8.2)	286.7 (178.8)	2.7 (0.7)	0.07 (0.02)	0.57 (0.36)	0.005 (0.00)
Non-AMF	26.0 (3.0)	84.3(57.7)	1.0 (0.6)	0.05 (0.01)	0.17 (0.12)	0.002 (0.00)
**t-values and significance**						
Treatment	1.0 ns	1.1ns	1.9 ns			

Note: Each treatment is 150, 1cm root segments (10 per slide) observed under a compound microscope. Means (n = 3) and (SE) are shown for each treatment.

ns: not significant

* p<0.05

** p<0.01

*** p<0.001

PAH concentrations (Fig 2) did not change significantly over the two years in the soil. In contrast, alkyl PAHs were shown to significantly increase in the soil from year 1 to year 2 (p = 0.006). Roots showed significant increase in both PAHs (p<0.001) and alkyl PAHs (p<0.0.05) from year 1 to year 2. Shoots showed no significant increase in PAHs (p = 0.08) or alkyl PAHs (p = 0.09) from year 1 to year 2.

**Fig 5:**
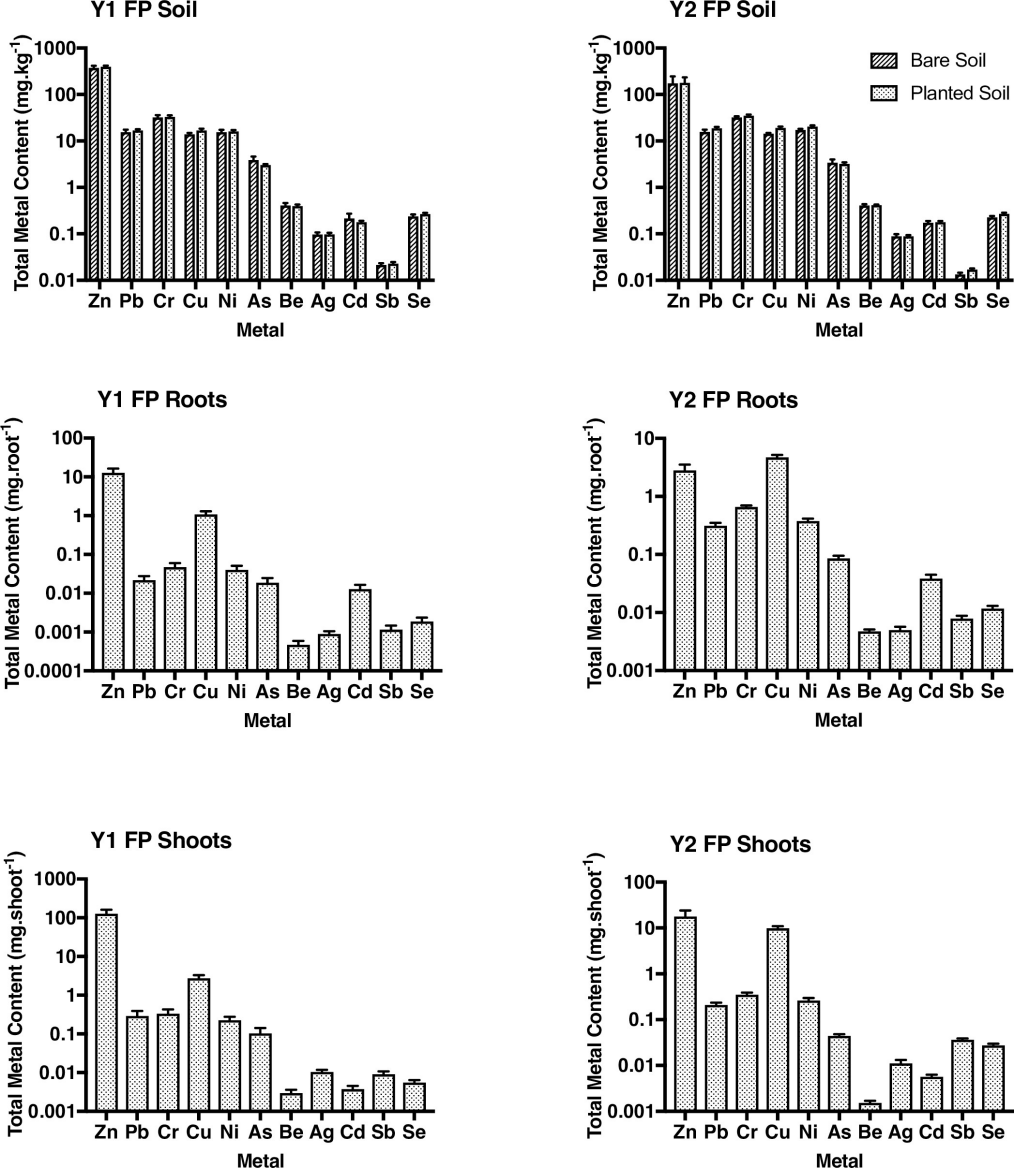
Mean (±SE) metal concentration (mg.kg^-1^) in soil and total metal content in roots (mg.root^-1^) and shoots (mg.shoot^-1^) of *E*. *purpurea*, from year 1 to year 2. Soil n = 5; roots and shoots, each n = 10.

The metal concentration in the field experiment soil showed increases in Ni (p<0.01) and decreases in Sb and Zn (p<0.001) from year 1 to year 2. (Fig 5). The roots showed significant increases in Ag, As, Be, Cd, Cr, Cu, Ni, Pb, Sb, Se (p<0.001), and Zn showed a significant decrease (p<0.05). In the shoots, Cu, Sb and Se significantly increased (p<0.001) as well as Cd (p<0.05). However, Be and Zn showed a significant decrease from year 1 to year 2 (p<0.05) in the shoots. (Fig 5).

## Discussion

*Roots* inoculated with *R*. *intraradices* showed increased PAH accumulation in the greenhouse study when compared with the non-inoculated treatment. This increase was particularly notable when measuring the root accumulation rates (*k*_1_) vs. log K_OW,_ AMF roots absorbing 10 x more PAHs than non-AMF roots. This increase was significant regardless of molecular weight or hydrophobicity in the soil. Increasing content of hydrocarbons in *E*. *purpurea* due to AMF inoculation can be explained through several mechanisms. The first mechanism is increased absorption in AMF inoculated roots of *E*. *purpurea*. AMF act as an extension of the roots and increase the surface area of the root system, making it more efficient for absorption of contaminants. The increased root surface area can enhance accumulation of contaminants with increasing soil concentrations of PAHs. In other studies, it has been shown that plant concentrations of PAHs generally increase with increasing soil PAH concentrations [40,41]. This holds true for our experiment when looking at PAHs. Another mechanism to explain the higher PAHs and alkyl PAHs in roots with AMF treatments could be the release of exudates from the roots or AMF. Exudates could be enhancing the bioavailability of PAHs, which was supported by previous findings [42–44]. The root exudates could cause a metabolic transformation of PAHs [45], using exo-enzymes to make them water-soluble [46]. By contrast to the greenhouse study, the field study showed no evidence of increased PAH root content with AMF pre-inoculation. However, based on accumulation rates, AMF treated roots showed greater accumulation of individual PAHs and this similar PAH root content could be explained by native AMF colonizing non-inoculated roots. Studies have shown that microorganisms in contaminated sites are highly efficient at accumulating and degrading soil contaminants [47,48].

Over the course of the greenhouse study, the soil carbon content remained relatively stable (3.30–3.75%) between treatments. However, in the field study, AMF and control soil had a decrease in organic carbon while non-AMF soil had almost a doubling in organic carbon, likely due to lower decomposition of organic matter from the previous summer. The decreasing pool of carbon in the soil matrix would increase the concentration of PAHs in the organic soil fraction, then reflecting a process known as “solvent depletion” [49]. This process may explain why higher rates of organic decomposition in soil results in higher PAHs and alkyl PAHs concentrations in residual soil carbon fractions, due to a depleting carbon pool in soil much like a distillation by solvent depletion. The AMF could be causing solvent depletion either directly, by breaking down soil C stores through root exudates or organic acids, or through stimulation of bacterial heterotrophs in the soil matrix.

In general, the soil concentrations of alkyl PAHs remained relatively constant over the course of both studies similarly to parent PAHs. However, alkyl PAHs accumulated more in plant tissues than PAHs, despite being less abundant in soils. This is the opposite of what was expected based on simple diffusion as well as other studies showing concentrations of PAHs in plants generally increase with increasing soil PAH concentrations [40,41]. Heitkamp and Cerniglia [50] found microbial degradation to be lower for alkyl PAHs than parent PAHs. Alternatively, exudates or enzymes being released from AMF could be increasing the bioavailability of the alkyl PAHs metabolically or co-metabolically [42,43,45,51] but have slow metabolic transformation or degradation by *E*. *purpurea*. This could explain why such high alkyl PAH content was found in the plant tissues, in both roots and shoots of both studies. AMF inoculation showed significantly higher accumulation in roots at week 20 in the greenhouse study, whilst alkyl PAHs increased significantly with time regardless of the treatment in the field study. The lack of differentiation between treatment groups for the field study could again be explained by native mycorrhizal colonization of all plant roots.

Our results on soil metal uptake by *E*. *purpurea* showed that metal concentrations and content were relatively unaffected by the presence or absence of the AMF, but were significantly affected by duration of the exposure in contaminated soil. The ‘enhanced uptake’ hypothesis [52,53], which predicts that AMF inoculated plants have a greater metal accumulation when compared to non-AMF inoculated plants is not supported in our study. This could be explained by the effect of native AMF, but not to the same degree as AMF pre-inoculated roots. Another possibility would be in line with other studies showing metals sequestered in the AMF tissues are prevented from transferring into the roots [32,47]. The AMF protection of plants is complimented by intrinsic strategies such as efflux pumping of metals that have entered the cytosol, use of metallothioneins for metal binding, and chelation of metals by organic acids and amino acids [14]. In further studies it would be beneficial to look at which exudates AMF are releasing into the contaminated soil, to determine what is driving the increased number of metal species being taken up by plants. Some of the exudates that increase in *E*. *purpurea* when inoculated with *R*. *intraradices* have been highlighted in a few studies looking into secondary metabolite production for plant defense. [54–56].

## Conclusions

The AMF inoculation was effective at increasing the accumulation of alkyl PAHs in the roots of *E*. *purpurea* as shown by 10-fold higher first order accumulation rate constants (K_1_) in the greenhouse study, however not significant under field conditions, likely because of native AMF present in the field site. Contaminants in plant material increased significantly from year 1 to year 2, indicating that long-term accumulation by plants has potential for soil bioremediation strategies. Plants may serve in bioremediation efforts to remove soil contaminants via harvesting, or to dilute soil contaminants via composting, though this study showed no evidence of soil contaminant dilution due to composting in the 2-year field study. In particular, alkyl PAHs showed preferential accumulation in plant tissues despite their lower concentrations in soils relative to parent PAHs, which will be especially useful in soils contaminated by petrogenic sources of PAHs, where alkyl PAHs predominate.

## Supporting information

S1 Fig Factorial block design used for the field plot. (DOCX)

S2 Fig Mean (±SE) hydrocarbon content (ng g-1) profile for from week 10 in the greenhouse study. n = 3.
(DOCX)

S3 Fig Mean (±SE) hydrocarbon content (ng g-1) profile for from week 20 in the greenhouse study. n = 3.
(DOCX)

S4 Fig Mean (±SE) hydrocarbon content (ng g-1) profile from year 1 (Y1) on Victoria Island, ON field site. n = 5.
(DOCX)

S5 Fig Mean (±SE) hydrocarbon content (ng g-1) profile from year 2 (Y2) on Victoria Island, ON field site. n = 5.
(DOCX)

S6 Fig Mean (±SE) metal content (mg kg-1) in soil samples of Echinacea purpurea in the greenhouse. CS (Control Soil), AMF (inoculated with Rhizoglomus intraradices), non-AMF (not inoculated), n = 5.
(DOCX)

S7 Fig Mean (±SE) metal content (mg kg-1) in soil samples of Echinacea purpurea in the field. Bare soil n = 5, Planted soil n = 10.
(DOCX)

S8 Fig Mean (±SE) metal content (mg) in root samples of Echinacea purpurea in the greenhouse. AMF (inoculated with Rhizoglomus intraradices), non-AMF (not inoculated), n = 5.
(DOCX)

S9 Fig Mean (±SE) metal content (mg) in root samples of Echinacea purpurea in the field n = 10. (DOCX)

S10 Fig Mean (±SE) metal content (mg) in shoot samples of Echinacea purpurea in the greenhouse. AMF (inoculated with Rhizoglomus intraradices), non-AMF (not inoculated), n = 5.
(DOCX)

S11 Fig Mean (±SE) metal content (mg) in shoot samples of Echinacea purpurea in the field. n = 10.
(DOCX)

S1 Table List of all compounds analyzed using GC-MS. (DOCX)

S1 File Data file. (XLSX)
