# Supplementary figures and images for: The accumulation of metals, PAHs and alkyl PAHs in the roots of Echinacea purpurea
Source: PLoS One. 2018 Dec 6;13(12):e0208325. doi: 10.1371/journal.pone.0208325 (PMC6283564; doi:10.1371/journal.pone.0208325)

**Factorial Block Design**


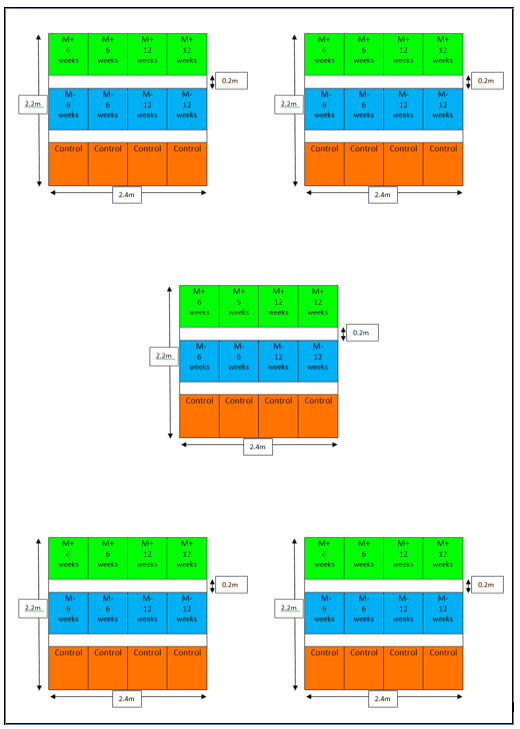


**S1 Figure.** Factorial block design used for the field plot.

Supplement: S1 Fig — (DOCX) [file pone.0208325.s001.docx]
