# Supplementary material for: The accumulation of metals, PAHs and alkyl PAHs in the roots of Echinacea purpurea
Source: PLoS One. 2018 Dec 6;13(12):e0208325. doi: 10.1371/journal.pone.0208325 (PMC6283564; doi:10.1371/journal.pone.0208325)
Supplement: S3 Fig — n = 3. (DOCX) [file pone.0208325.s003.docx]

**S3 Figure.** Mean (±SE) hydrocarbon content (ng g^-1^ dry weight) profile for from week 20 in the greenhouse study. n=3.
