# Supplementary material for: The accumulation of metals, PAHs and alkyl PAHs in the roots of Echinacea purpurea
Source: PLoS One. 2018 Dec 6;13(12):e0208325. doi: 10.1371/journal.pone.0208325 (PMC6283564; doi:10.1371/journal.pone.0208325)
Supplement: S4 Fig — n = 5. (DOCX) [file pone.0208325.s004.docx]

**S4 Figure.** Mean (±SE) hydrocarbon content (ng g^-1^dry weight) profile from year 1 (Y1) on Victoria Island, ON field site. n=5.
