# Supplementary material for: The accumulation of metals, PAHs and alkyl PAHs in the roots of Echinacea purpurea
Source: PLoS One. 2018 Dec 6;13(12):e0208325. doi: 10.1371/journal.pone.0208325 (PMC6283564; doi:10.1371/journal.pone.0208325)
Supplement: S7 Fig — Bare soil n = 5, Planted soil n = 10. (DOCX) [file pone.0208325.s007.docx]

**S7 Figure.** Mean (±SE) metal content (mg kg^-1^) in soil samples of *Echinacea purpurea in* the field. Bare soil n=5, Planted soil n=10.
