# Supplementary material for: The accumulation of metals, PAHs and alkyl PAHs in the roots of Echinacea purpurea
Source: PLoS One. 2018 Dec 6;13(12):e0208325. doi: 10.1371/journal.pone.0208325 (PMC6283564; doi:10.1371/journal.pone.0208325)
Supplement: S9 Fig — (DOCX) [file pone.0208325.s009.docx]

**S9 Figure.** Mean (±SE) metal content (mg) in root samples of *Echinacea purpurea in* the field n=10.
