# Supplementary material for: The accumulation of metals, PAHs and alkyl PAHs in the roots of Echinacea purpurea
Source: PLoS One. 2018 Dec 6;13(12):e0208325. doi: 10.1371/journal.pone.0208325 (PMC6283564; doi:10.1371/journal.pone.0208325)
Supplement: S10 Fig — AMF (inoculated with Rhizoglomus intraradices), non-AMF (not inoculated), n = 5. (DOCX) [file pone.0208325.s010.docx]

**S10 Figure.** Mean (±SE) metal content (mg) in shoot samples of *Echinacea purpurea in* the greenhouse. AMF (inoculated with *Rhizoglomus intraradices*), non-AMF (not inoculated), n=5.
