# Supplementary material for: The accumulation of metals, PAHs and alkyl PAHs in the roots of Echinacea purpurea
Source: PLoS One. 2018 Dec 6;13(12):e0208325. doi: 10.1371/journal.pone.0208325 (PMC6283564; doi:10.1371/journal.pone.0208325)
Supplement: S1 Table — (DOCX) [file pone.0208325.s012.docx]

**S1 Table.** List of all compounds analyzed using GC-MS.

| Type | PAH Compound | IUPAC | CAS # | Structure |
| --- | --- | --- | --- | --- |
| Low Molecular Weight (LMW) | Naphthalene |  | 91-20-3 | 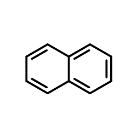 |
|  | Acenaphthylene |  | 208-96-8 | 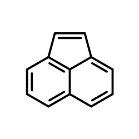 |
|  | Acenaphthene |  | 83-32-9 | 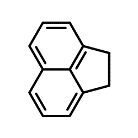 |
|  | Fluorene |  | 86-73-7 | 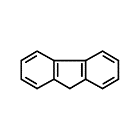 |
|  | Phenanthrene |  | 85-01-8 | 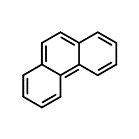 |
|  | Anthracene |  | 120-12-7 | 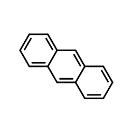 |
|  | Fluoranthene |  | 206-44-0 | 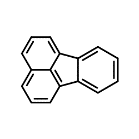 |
|  |  |  |  |  |
| High Molecular Weight (HMW) | Pyrene |  | 129-00-0 | 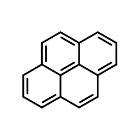 |
|  | Benz[a]anthracene | tetraphene | 56-55-3 | 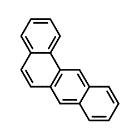 |
|  | Chrysene |  | 218-01-9 | 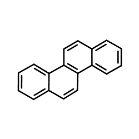 |
|  | Benzo[b]fluoranthene |  | 205-99-2 | 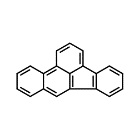 |
|  | Benzo[k]fluoranthene |  | 207-08-9 | 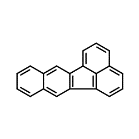 |
|  | Benzo[a]pyrene | Benzo[pqr]tetraphene | 50-32-8 | 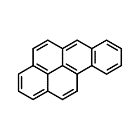 |
|  | Indeno[1,2,3-cd]pyrene | IP | 193-39-5 | 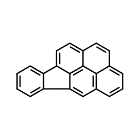 |
|  | Dibenz[a,h]anthracene | Benzo[k]tetraphene | 53-70-3 | 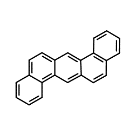 |
|  | Benzo[g,h,i]perylene |  | 191-24-2 | 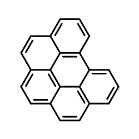 |
|  |  |  |  |  |
| Alkyl PAHs | C2-Naphthalene |  |  |  |
|  | C3-Naphthalene |  |  |  |
|  | C4-Naphthalene |  |  |  |
|  |  |  |  |  |
|  | C1-Fluorene |  |  |  |
|  | C2-Fluorene |  |  |  |
|  | C3-Fluorene |  |  |  |
|  |  |  |  |  |
|  | C1-Phenanthrene/Anthracene |  |  |  |
|  | C2-Phenanthrene/Anthracene |  |  |  |
|  | C3-Phenanthrene/Anthracene |  |  |  |
|  | C4-Phenanthrene/Anthracene |  |  |  |
|  |  |  |  |  |
|  | C1-Dibenzothiophenes |  |  |  |
|  | C2-Dibenzothiophenes |  |  |  |
|  | C3-Dibenzothiophenes |  |  |  |
|  | C4-Dibenzothiophenes |  |  |  |
|  |  |  |  |  |
|  | C1-Fluoranthene/Pyrene |  |  |  |
|  | C2-Fluoranthene/Pyrene |  |  |  |
|  | C3-Fluoranthene/Pyrene |  |  |  |
|  | C4-Fluoranthene/Pyrene |  |  |  |
|  |  |  |  |  |
|  | C1-Benz[a]anthracene/Chrysene |  |  |  |
|  | C2-Benz[a]anthracene/Chrysene |  |  |  |
|  | C3-Benz[a]anthracene/Chrysene |  |  |  |
|  | C4-Benz[a]anthracene/Chrysene |  |  |  |
|  |  |  |  |  |
|  | C1-Benzofluoranthene/Benzopyrene |  |  |  |
|  | C2-Benzofluoranthene/Benzopyrene |  |  |  |
